# Air Pollution and Respiratory Infections during Early Childhood: An Analysis of 10 European Birth Cohorts within the ESCAPE Project

**DOI:** 10.1289/ehp.1306755

**Published:** 2013-10-22

**Authors:** Elaina A. MacIntyre, Ulrike Gehring, Anna Mölter, Elaine Fuertes, Claudia Klümper, Ursula Krämer, Ulrich Quass, Barbara Hoffmann, Mireia Gascon, Bert Brunekreef, Gerard H. Koppelman, Rob Beelen, Gerard Hoek, Matthias Birk, Johan C. de Jongste, H.A. Smit, Josef Cyrys, Olena Gruzieva, Michal Korek, Anna Bergström, Raymond M. Agius, Frank de Vocht, Angela Simpson, Daniela Porta, Francesco Forastiere, Chiara Badaloni, Giulia Cesaroni, Ana Esplugues, Ana Fernández-Somoano, Aitana Lerxundi, Jordi Sunyer, Marta Cirach, Mark J. Nieuwenhuijsen, Göran Pershagen, Joachim Heinrich

**Affiliations:** 1 Institute of Epidemiology I, Helmholtz Zentrum München, German Research Centre for Environmental Health, Munich, Germany; 2 Institute for Risk Assessment Sciences, Utrecht University, Utrecht, the Netherlands; 3 Centre for Epidemiology, Institute of Population Health, Manchester Academic Health Sciences Centre, The University of Manchester, Manchester, United Kingdom; 4 School of Population and Public Health, University of British Columbia, Vancouver, British Columbia, Canada; 5 IUF-Leibniz Research Institute for Environmental Medicine, University of Düsseldorf, Düsseldorf, Germany; 6 Air Quality & Sustainable Nanotechnology, IUTA (Institut für Energie-und Umwelttechnik e.V.), Duisburg, Germany; 7 Medical Faculty, Heinrich Heine University Düsseldorf, Düsseldorf, Germany; 8 Centre for Research in Environmental Epidemiology (CREAL), Barcelona, Catalonia, Spain; 9 Spanish Consortium for Research on Epidemiology and Public Health (CIBERESP), Spain; 10 Julius Center for Health Sciences and Primary Care, University Medical Center Utrecht, Utrecht, the Netherlands; 11 Department of Pediatric Pulmonology and Pediatric Allergology, Beatrix Children’s Hospital, and; 12 GRIAC (Groningen Research Institute of Asthma and COPD), University of Groningen, University Medical Center Groningen, Groningen, the Netherlands; 13 Department of Pediatrics, Division of Respiratory Medicine, Erasmus University Medical Center/Sophia Children’s Hospital, Rotterdam, the Netherlands; 14 Institute of Epidemiology II, Helmholtz Zentrum München, German Research Centre for Environmental Health, Munich, Germany; 15 Institute of Environmental Medicine, Karolinska Institutet, Stockholm, Sweden; 16 Institute of Inflammation and Repair, Manchester Academic Health Sciences Centre, The University of Manchester, Manchester, United Kingdom; 17 Department of Epidemiology, Lazio Regional Health Service ASL RME (Dipartimento di epidemiologia ASL Roma E), Rome, Italy; 18 Centre of Public Health Research (CSISP), Valencia, Spain; 19 Department of Preventive Medicine, University of Oviedo, Asturias, Spain; 20 BioDonostia Health Research Institute, Donostia, Spain; 21 Department of Public Health and Preventive Medicine, University of Basque Country, Bilbão, Spain; 22 Hospital del Mar Medical Research Institute (IMIM), Barcelona, Catalonia, Spain; 23 Pompeu Fabra University, Department of Experimental and Health Sciences, Barcelona, Catalonia, Spain

## Abstract

Background: Few studies have investigated traffic-related air pollution as a risk factor for respiratory infections during early childhood.

Objectives: We aimed to investigate the association between air pollution and pneumonia, croup, and otitis media in 10 European birth cohorts—BAMSE (Sweden), GASPII (Italy), GINIplus and LISAplus (Germany), MAAS (United Kingdom), PIAMA (the Netherlands), and four INMA cohorts (Spain)—and to derive combined effect estimates using meta-analysis.

Methods: Parent report of physician-diagnosed pneumonia, otitis media, and croup during early childhood were assessed in relation to annual average pollutant levels [nitrogen dioxide (NO_2_), nitrogen oxide (NO_x_), particulate matter ≤ 2.5 μm (PM_2.5_), PM_2.5_ absorbance, PM_10_, PM_2.5–10_ (coarse PM)], which were estimated using land use regression models and assigned to children based on their residential address at birth. Identical protocols were used to develop regression models for each study area as part of the ESCAPE project. Logistic regression was used to calculate adjusted effect estimates for each study, and random-effects meta-analysis was used to calculate combined estimates.

Results: For pneumonia, combined adjusted odds ratios (ORs) were elevated and statistically significant for all pollutants except PM_2.5_ (e.g., OR = 1.30; 95% CI: 1.02, 1.65 per 10-μg/m^3^ increase in NO_2_ and OR = 1.76; 95% CI: 1.00, 3.09 per 10-μg/m^3^ PM_10_). For otitis media and croup, results were generally null across all analyses except for NO_2_ and otitis media (OR = 1.09; 95% CI: 1.02, 1.16 per 10-μg/m^3^).

Conclusion: Our meta-analysis of 10 European birth cohorts within the ESCAPE project found consistent evidence for an association between air pollution and pneumonia in early childhood, and some evidence for an association with otitis media.

Citation: MacIntyre EA, Gehring U, Mölter A, Fuertes E, Klümper C, Krämer U, Quass U, Hoffmann B, Gascon M, Brunekreef B, Koppelman GH, Beelen R, Hoek G, Birk M, de Jongste JC, Smit HA, Cyrys J, Gruzieva O, Korek M, Bergström A, Agius RM, de Vocht F, Simpson A, Porta D, Forastiere F, Badaloni C, Cesaroni G, Esplugues A, Fernández-Somoano A, Lerxundi A, Sunyer J, Cirach M, Nieuwenhuijsen MJ, Pershagen G, Heinrich J. 2014. Air pollution and respiratory infections during early childhood: an analysis of 10 European birth cohorts within the ESCAPE project. Environ Health Perspect 122:107–113; http://dx.doi.org/10.1289/ehp.1306755

## Introduction

Respiratory infections are a leading reason for outpatient physician visits and hospitalizations among children (Williams et al. 2002). Most infections resolve with minimal use of health care resources; however, episodes of severe or recurrent infection may require hospitalization or surgery, and the resultant burden on resources is substantial (Black et al. 2010).

Young children are particularly susceptible to respiratory pathogens and also to air pollution (Bateson and Schwartz 2008; Heinrich and Slama 2007). There is strong evidence that indoor air pollution, such as secondhand smoke and the use of biomass, is a risk factor for respiratory infections in children (da Costa et al. 2004). Evidence is growing to support an association with outdoor air pollution as well (Brauer et al. 2006; Leonardi et al. 2000; MacIntyre et al. 2011).

The European Study of Cohorts for Air Pollution Effects (ESCAPE 2009) is a project aimed at investigating the impacts of long-term exposure to air pollution through the development of harmonized exposure data assigned to previously established cohorts that have collected information on specific health outcomes of interest for air pollution research. We analyzed data from 10 European birth cohorts and completed a meta-analysis of air pollution and respiratory infection (pneumonia, croup, and otitis media) during early childhood.

## Methods

*Study population*. We included 10 ESCAPE birth cohorts. The inclusion criteria for each birth cohort were that data on at least one outcome of interest were available during early childhood, and that the ESCAPE exposure assignment was complete.

BAMSE (Children, Allergy, Milieu, Stockholm, Epidemiological Survey) is a population-based prospective birth cohort of children born during 1994–1996 in Stockholm County, Sweden (Wickman et al. 2002). GASPII (Gene and Environment: Prospective Study on Infancy in Italy) is a prospective birth cohort of children born during 2003–2004 in Rome, Italy (Porta et al. 2007). GINIplus (German Infant Nutrition Intervention Study Plus environmental and genetic influences on allergy development) is a population-based prospective birth cohort, with a nutritional intervention, of children born during 1995–1998 in Wesel and Munich, Germany (Zirngibl et al. 2002). LISAplus (Influence of Life-style Factors on the Development of the Immune System and Allergies in Childhood Plus the influence of traffic emissions and genetics) is a population-based prospective birth cohort study of children born during 1997–1999 in Wesel, Munich, Leipzig, and Bad Honnef, Germany (Heinrich et al. 2002). INMA (INfancia y Medio Ambiente; Environment and Childhood) is a network of Spanish birth cohorts. The four INMA cohorts in the present analysis comprise children born during 2004–2008 in both major cities and rural towns—Asturias, Gipuzkoa, Sabadell, and Valencia (Guxens et al. 2012). MAAS (Manchester Asthma and Allergy Study) is an unselected, prospective population-based birth cohort study (with a small nested allergen control intervention) of children born during 1995–1997 in the Greater Manchester conurbation in the United Kingdom (Custovic et al. 2002). Finally, the PIAMA (Prevention and Incidence of Asthma and Mite Allergy) study is a population based prospective birth cohort, with an intervention component, of children born during 1996–1997 in cities and small towns across the Netherlands (Brunekreef et al. 2002). Each cohort obtained parental consent and protocols were approved by all relevant ethical review boards.

Exposure to outdoor air pollution was estimated using land use regression (LUR) models, and methods were harmonized across each cohort (Beelen et al. 2013; Cyrys et al. 2012; Eeftens et al. 2012a, 2012b; ESCAPE 2013). Briefly, sampling sites for particulate matter (*n* = 20–40) and nitrogen oxides (*n* = 40–80) were strategically chosen for each study area to represent the spatial distribution of concentrations at the residential address of each child at birth, with some overrepresentation of locations with heavy traffic where the largest heterogeneity was expected. Three 2-week sampling campaigns were spread out over 1 year and used to estimate an annual average. Measurement data used to develop the LUR models were collected during 2008–2009 (BAMSE, GINIplus/LISAplus, PIAMA), 2009 (INMA), 2009–2010 (MAAS), and 2010–2011 (GASPII). The annual average was temporally adjusted using continuous measurement data from a fixed monitor that was used to capture background levels in each study area. Models based on GIS (geographic information system) variables related to traffic, land use, population density, altitude, and regional background pollution were developed using common ESCAPE protocols to predict measured air pollution concentrations.

Separate models were developed for nitrogen dioxide (NO_2_), nitrogen oxides [NO_x_ (NO_2_ + NO)], PM_2.5_ (particulate matter ≤ 2.5 μm in diameter), PM_2.5_ absorbance, PM_10_ (≤ 10 μm in diameter), and coarse PM (PM_10_–PM_2.5_). In addition, two variables were created to describe traffic intensity at the residential address: traffic intensity on the nearest street, and traffic load on all major roads within a 100-m buffer. Annual average air pollution concentrations and traffic intensity variables were assigned to children for the first year of life based on their residential address reported at birth. The LISAplus study centers of Leipzig and Bad Honnef were not included in the ESCAPE exposure assessment, so children from these cities could not be included in the meta-analysis.

The air pollution data used to derive the ESCAPE exposure models were measured in 2008–2011, whereas children included in the study cohorts were born as early as 1994. Therefore, we conducted sensitivity analyses using routine monitoring data to back-extrapolate exposure estimates based on LUR to each child’s year of birth. We used two approaches for the back-extrapolation: The first used the ratio of the average concentration measured from the date of birth through the second birthday to the average concentration measured during the ESCAPE monitoring year; the second used the absolute difference between the average concentrations at each time period (ESCAPE 2013). Both methods altered the spatial contrast derived from the current LUR models without affecting the spatial patterns of air pollutants in the study areas.

For each cohort, parents reported (yes/no) physician-diagnosed pneumonia, otitis media, and croup during early childhood (see Supplemental Material, p. 2, for the specific questions used for each cohort). Outcomes were assessed at 6 months (GASPII, LISAplus), 1 year (BAMSE, GINIplus, INMA Valencia, LISAplus, PIAMA), 14 months (INMA Gipuzkoa, INMA Sabadell), 15 months (GASPII), 18 months (LISAplus, INMA Asturias), 2 years (BAMSE, GINIplus, LISAplus, PIAMA), and 3 years (MAAS). It was not possible to evaluate respiratory infections restricted to the first 2 years of life for the MAAS birth cohort because these outcomes were not assessed in the full cohort until 3 years of age. Pneumonia data were available for all cohorts; otitis media data were available for all except GINIplus and MAAS; and croup data were available for all except GASPII, the four INMA cohorts, and PIAMA. Cumulative incidence was modeled in each analysis, unless otherwise specified.

We used logistic binomial regression in all individual cohort analyses, and statistical significance was defined by *p*-values < 0.05. Air pollution was entered as a continuous variable and was not transformed. Models were assessed using the Hosmer–Lemeshow goodness-of-fit test and the Pearson’s chi-square test. Potential confounders were identified from previous literature and selected *a priori*. Individual cohort models were adjusted for municipality/city (BAMSE only), sex, older siblings (any/none), partial or exclusive breastfeeding at 6 months, atopy of either parent, child-care attendance reported at any time during follow-up, maternal smoking during pregnancy, secondhand smoke in the home reported at any time during follow-up (not available for INMA), visible mold or dampness in the home, use of gas stove, birth season (winter: January–March; spring: April–June; summer: July–September; fall: October–December), parental socioeconomic status [highest education attained by either parent (BAMSE, GINIplus, LISAplus, PIAMA, INMA: low, medium, high); highest occupational level by either parent (GASPII: low, medium, high); or household income (MAAS: < £10,000; £10,000–20,000; £20,000–30,000; > £30,000)], and intervention (GINIplus, MAAS, and PIAMA only). Models for traffic intensity and traffic load were additionally adjusted for background NO_2_ concentrations. Children with missing data for any covariate were excluded from individual analyses. Based on the ESCAPE protocol, we calculated estimates for the following increments in exposure: 10 μg/m^3^ for NO_2_, 20 μg/m^3^ for NO_x_, 1 unit for PM_2.5_ absorbance, 5 μg/m^3^ for PM_2.5_, 10 μg/m^3^ for PM_10_, 5 μg/m^3^ for coarse PM, 5,000 vehicles/day for traffic intensity on the nearest street; and 4,000 vehicle-km/day for traffic load on major roads within a 100-m buffer. We assessed heterogeneity of effect estimates between studies using the *I*^2^ statistic (Huedo-Medina et al. 2006). We used random-effects meta-analysis models to calculate combined estimates (DerSimonian and Laird 1986).

We used sensitivity analyses to test the robustness of effect estimates to the inclusion of additional potential confounders: birth weight, maternal age at birth, and area-level socioeconomic indicators. In addition, we stratified associations for outcomes that were diagnosed during the first year of life and outcomes diagnosed during the second year of life for cohorts that completed follow-ups at 1 and 2 years of age (BAMSE, GINIplus, LISAplus, PIAMA). Additional analyses were stratified by sex, parental socioeconomic status (low, middle, or high), and residential mobility (moved from the birth address at any time during the follow-up period) to examine potential effect modification. As noted above, we also performed sensitivity analyses using exposure estimates that were recalculated for selected pollutants using back-extrapolation techniques to assess the consistency of associations. In addition, we performed a sensitivity analysis of the influence of neighborhood clustering by including an area-level variable (BAMSE: neighborhood; GINIplus: ZIP code; LISAplus: ZIP code; INMA: rural indicator; PIAMA: neighborhood) as a random effect in adjusted models. Area-level data were not available for GASPII or MAAS. Finally, we used two-pollutant models to estimate the independent effects of NO_2_ and PM.

All individual and combined analyses were completed using identical protocols. Individual estimates are presented by cohort except for the German birth cohorts (LISAplus and GINIplus), which had almost identical study designs and parental questionnaires, and are presented as GINI/LISA North (Wesel) and GINI/LISA South (Munich) because separate air pollution models were developed for each area as part of ESCAPE. Statistical analyses were completed using SPSS version 20 (IBM SPSS, Armonk, NY, USA) and SAS version 9.1 (SAS Institute Inc., Cary NC, USA).

## Results

There was complete outcome (at least one), exposure (a minimum of NO_2_ and NO_x_) and potential confounder information for 16,059 children across all 10 cohorts (79.6% of the total recruited population). Children excluded due to missing data were more likely to have parents of lower socioeconomic status (BAMSE, GINI/LISA South, GINI/LISA North, MAAS, PIAMA), mothers who smoked during pregnancy (BAMSE, GASPII, GINI/LISA South, GINI/LISA North, PIAMA); and were less likely to be breastfed for at least 6 months (GASPII, GINI/LISA South, GINI/LISA North, MAAS, PIAMA) or to have atopic parents (GINI/LISA South, INMA Sabadell). Table 1 shows the cumulative incidence of parent-reported physician-diagnosed respiratory infection, by cohort. The cumulative incidence of pneumonia during early childhood ranged from 1.5% in INMA Sabadell to 7.9% in BAMSE (0.7–3.6% during the first year only). Otitis media ranged from 21.8% in GASPII to 50.0% in BAMSE (6.8–26.6% for the first year), and croup ranged from 10.6% in MAAS to 12.9% in GINI/LISA North (4.2–5.6% for the first year). There were differences in breastfeeding, child-care attendance, parental atopy, and secondhand smoke exposure among the cohorts (see Supplemental Material, Table S1). Air pollution concentrations were highest in GASPII and lowest in BAMSE; GINI/LISA South, GINI/LISA North and PIAMA had similar mean concentrations (Table 1). Additional statistics on air pollutant concentrations by cohort are available in Supplemental Material, Table S2. Air pollutant concentrations were moderately to highly correlated (see Supplemental Material, Table S3; e.g., correlation between PM_2.5_ and NO_2_ ranged between 0.42 and 0.80, and correlations between PM_2.5_ absorbance and NO_2_ ranged between 0.40 and 0.93).

**Table 1:** The cumulative incidence of respiratory infections and distribution of air pollution for each ESCAPE birth cohort.

	BAMSE	GASPII	GINI/LISA South	GINI/LISA North	INMA Asturias	INMA Gipuzkoa	INMA Sabadell	INMA Valencia	MAAS	PIAMA
Respiratory infections [*n* (%)]
Total included	3,821 (100)	678 (100)	3,321 (100)	2,460 (100)	360 (100)	437 (100)	402 (100)	559 (100)	695 (100)	3,475 (100)
Pneumonia, 0–1 year	137 (3.6)	5 (0.7)^*a*^	81 (2.4)	80 (3.3)	—	—	—	10 (1.8)^*b*^	—	84 (2.4)
Pneumonia, 0–2 years	301 (7.9)	14 (2.1)^*c*^	198 (6.0)	144 (5.9)	8 (2.2)^*d*^	9 (2.1)^*e*^	6 (1.5)^*e*^	—	13 (1.9)^*f*^	150 (4.3)
Otitis media, 0–1 year	1,017 (26.6)^*g*^	46 (6.8)^*a*^	202(16.4)^*h*^	49 (18.2)^*h*^	—	—	—	139 (24.9)^*b*^	—	603 (18.0)
Otitis media, 0–2 years	1,911 (50.0)^*g*^	148 (21.8)^*c*^	422 (34.2)^*h*^	103 (38.3)^*h*^	143 (39.7)^*d*^	161 (36.8)^*e*^	144 (35.8)^*e*^	—	—	1,144 (32.9)
Croup, 0–1 year	201 (5.3)	—	140 (4.2)	135 (5.6)	—	—	—	—	—	—
Croup, 0–2 years	410 (10.7)	—	362 (10.9)	318 (12.9)	—	—	—	—	74 (10.6)^*f*^	—
Air pollution (median [IQR])
NO_2_ (μg/m^3^)	12.4 (9.3)	43.2 (10.4)	20.8 (8.3)	23.2 (3.2)	22.2 (14.4)	18.4 (5.7)	41.7 (12.4)	27.9 (18.1)	23.0 (2.4)	23.1 (8.4)
NO_x_ (μg/m^3^)	20.9 (18.2)	65.8 (23.9)	34.6 (12.2)	33.3 (8.4)	44.0 (39.0)	37.2 (11.1)	69.7 (21.2)	44.7 (31.1)	38.7 (5.2)	32.8 (11.0)
PM_2.5_ (μg/m^3^)	8.1 (1.9)	18.8 (2.0)	13.3 (1.2)	17.2 (0.9)	—	—	14.6 (1.1)	—	9.4 (0.0)	16.5 (1.2)
PM_2.5_ absorbance (10^–5^/m)	0.6 (0.3)	2.5 (0.4)	1.7 (0.2)	1.2 (0.2)	—	—	2.2 (0.5)	—	1.1 (0.2)	1.2 (0.3)
PM_10_ (μg/m^3^)	15.6 (3.9)	34.9 (6.1)	20.4 (2.9)	25.2 (1.6)	—	—	26.4 (3.4)	—	17.0 (0.2)	24.6 (1.2)
Coarse PM (μg/m^3^)	7.7 (3.0)	15.7 (4.3)	6.5 (2.0)	8.4 (0.7)	—	—	11.3 (2.5)	—	6.9 (0.8)	8.1 (0.8)
Traffic intensity on nearest street (vehicles/day)	500 (1,450)	—	500 (0)	500 (0)	—	—	—	—	500 (0)	215 (436)
Traffic load on major streets within 100-m buffer (vehicle × m/day)	0 (1,621,333)	—	0 (1,318,179)	0 (0)	—	—	—	—	0 (0)	0 (0)
IQR, interquartile range.^***a***^Assessed at 6 months. ^***b***^Assessed at 12 months. ^***c***^Assessed at 15 months. ^***d***^Assessed at 18 months. ^***e***^Assessed at 14 months. ^***f***^Assessed at 3 years. ^***g***^Requiring antibiotic. ^***h***^Otitis media was collected only in the LISAplus study (total South *n* = 1,242; North *n* = 280).										

**Figure 1:**
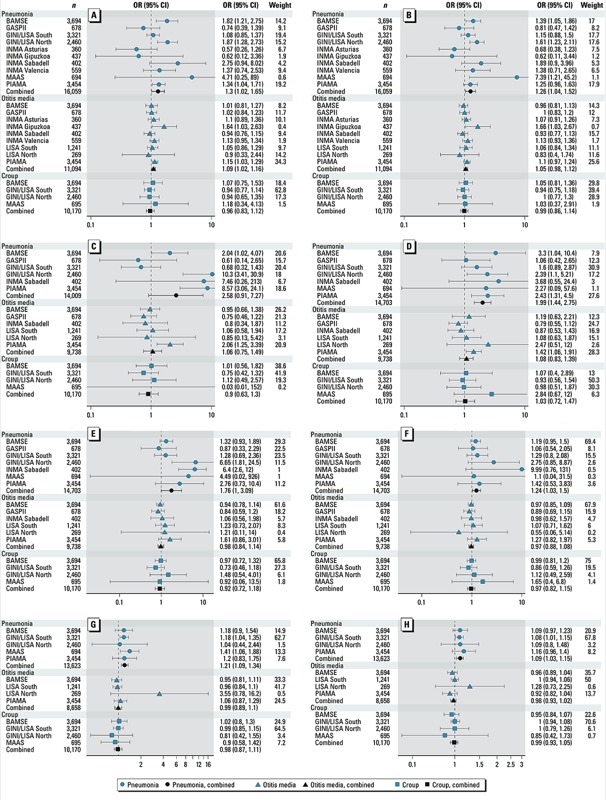
Forest plots of individual cohort and combined effect estimates (ORs) by outcome for (*A*) NO_2_, (*B*) NO_x_, (*C*) PM_2.5_, (*D*) PM_2.5_ absorbance, (*E*) PM_10_, (*F*) coarse PM, (*G*) traffic intensity on nearest street, and (*H*) traffic load on all major roads. Weight indicates relative weight (%) assigned using random-effects meta-analysis. The lifetime cumulative incidence of respiratory infection (pneumonia, otitis media, croup) was assessed at 12 months (INMA Valencia), 14 months (INMA Gipuzkoa, INMA Sabadell), 15 months (GASPII), 18 months (INMA Asturias), 24 months (BAMSE, GINI/LISA North, GINI/LISA South, PIAMA), and 36 months (MAAS) of age. Individual cohort models were adjusted for municipality (BAMSE), sex, older siblings, breastfeeding at 6 months, atopy of either parent, any child-care reported during follow-up, maternal smoking during pregnancy, any environmental tobacco smoke in the child’s home reported during follow-up, visible mold or dampness in the home, use of gas stove, birth season, parental socioeconomic status (low, medium, high), and intervention (GINIplus, MAAS, PIAMA). Associations are presented for the following increments in exposure: 10 μg/m^3^ for NO_2_, 20 μg/m^3^ for NO_x_, 5 μg/m^3^ for PM_2.5_, 1 unit for PM_2.5_ absorbance, 10 μg/m^3^ for PM_10_, 5 μg/m^3^ for coarse PM, 5,000 vehicles/day for traffic intensity on the nearest street; and 4,000 vehicle-km/day for traffic load on major roads within a 100-m buffer.

Associations between air pollution and respiratory infection during early childhood are presented in Figure 1 for *a*) individual and *b*) combined effect estimates. Table 2 presents combined effect estimates for crude (adjusted for sex and municipality) and adjusted (adjusted for all potential confounders) models and *p*-values for heterogeneity. The heterogeneity between studies varied and the largest *I*^2^ statistics were for models of pneumonia and NO_2_, PM_2.5_, and PM_10_. Effect estimates were robust to adjustment for older siblings, breastfeeding, parental atopy, child care, maternal smoking during pregnancy, environmental tobacco smoke, visible mold or dampness, use of gas stove, birth season, and parental socioeconomic status. For pneumonia, elevated odds ratios (ORs) were found in almost all analyses, and the combined estimates were statistically significant for all measures of air pollution except PM_2.5_ (OR = 2.58; 95% CI: 0.91, 7.27 for a 5-μg/m^3^ increase). For otitis media and croup, results were generally null across all analyses except for NO_2_ and otitis media, for which the adjusted OR was 1.09 (95% CI: 1.02, 1.16 for a 10-μg/m^3^ increase).

**Table 2:** Combined estimates from random-effects meta-analyses for residential air pollution and respiratory infections during early life (up to 36 months).^*a*^

	Crude^*b*^			Adjusted^*c*^		
	OR (95% CI)	*I*^2^	*p*-Value	OR (95% CI)	*I*^2^	*p*-Value
Pneumonia
NO_2_	1.25 (1.04, 1.50)*	37.1	0.112	1.30 (1.02, 1.65)*	52.9	0.024
NO_x_	1.23 (1.06, 1.41)*	22.2	0.239	1.26 (1.04, 1.52)*	44.0	0.066
PM_2.5_	2.13 (0.82, 5.49)	79.7	0.000	2.58 (0.91, 7.27)	81.7	0.000
PM_2.5_ absorbance	1.78 (1.30, 2.43)*	0	0.734	1.99 (1.44, 2.75)*	0	0.663
PM_10_	1.55 (1.03, 2.34)*	29.2	0.205	1.76 (1.00, 3.09)*	51.2	0.051
Coarse PM	1.23 (1.02, 1.47)*	0	0.626	1.24 (1.03, 1.50)*	0	0.579
Traffic, nearest street	1.08 (1.03, 1.14)*	0	0.997	1.09 (1.03, 1.15)*	0	0.969
Traffic, major streets	1.19 (1.08, 1.31)*	0	0.979	1.21 (1.09, 1.34)*	0	0.843
Otitis media
NO_2_	1.08* (1.01, 1.15)	4.8	0.395	1.09 (1.02, 1.16)*	0	0.515
NO_x_	1.04 (0.98, 1.10)	0.5	0.430	1.05 (0.98, 1.12)	0	0.458
PM_2.5_	1.02 (0.71, 1.45)	55.5	0.047	1.06 (0.75, 1.49)	47.9	0.088
PM_2.5_ absorbance	1.05 (0.80, 1.37)	46.7	0.095	1.08 (0.83, 1.39)	39.9	0.139
PM_10_	0.98 (0.83, 1.17)	11.6	0.341	0.98 (0.84, 1.14)	0	0.539
Coarse PM	0.96 (0.87, 1.06)	0	0.608	0.97 (0.88, 1.08)	0	0.805
Traffic, nearest street	0.98 (0.94, 1.03)	1.4	0.385	0.98 (0.93, 1.02)	0	0.497
Traffic, major streets	1.00 (0.91, 1.09)	0	0.462	0.99 (0.89, 1.10)	18.2	0.300
Croup
NO_2_	0.92 (0.80, 1.07)	0	0.884	0.96 (0.83, 1.12)	0	0.909
NO_x_	0.96 (0.83, 1.10)	0	0.895	0.99 (0.86, 1.14)	0	0.936
PM_2.5_	0.83 (0.58, 1.19)	0	0.760	0.90 (0.63, 1.30)	0	0.703
PM_2.5_ absorbance	0.95 (0.66, 1.37)	5.0	0.368	1.03 (0.72, 1.47)	0	0.554
PM_10_	0.89 (0.70, 1.13)	0	0.586	0.92 (0.72, 1.18)	0	0.595
Coarse PM	0.95 (0.80, 1.12)	0	0.551	0.97 (0.82, 1.15)	0	0.787
Traffic, nearest street	0.98 (0.93, 1.04)	0	0.926	0.99 (0.93, 1.05)	0	0.853
Traffic, major streets	0.97 (0.86, 1.09)	0	0.734	0.98 (0.87, 1.11)	0	0.901
Associations are presented for the following increments in exposure: 10 μg/m^3^ for NO_2_, 20 μg/m^3^ for NO_x_, 5 μg/m^3^ for PM_2.5_, 1 unit for PM_2.5_ absorbance, 10 μg/m^3^ for PM_10_, 5 μg/m^3^ for coarse PM, 5,000 vehicles/day for traffic intensity on the nearest street; and 4,000 vehicle-km/day for traffic load on major roads within a 100-m buffer; associations with traffic intensity and traffic load were additionally adjusted for background NO_2_ concentrations.^***a***^Outcomes assessed up to 12 months (INMA Valencia), 14 months (INMA Gipuzkoa, INMA Sabadell), 15 months (GASPII), 18 months (INMA Asturias), 24 months (BAMSE, GINIplus, LISAplus, PIAMA), and 36 months (MAAS). ^***b***^Crude models were adjusted for sex and municipality (BAMSE). ^***c***^Adjusted models included municipality (BAMSE), sex, older siblings, breastfeeding at 6 months, atopy of either parent, any child-care reported during follow-up, maternal smoking during pregnancy, any environmental tobacco smoke in the child’s home reported during follow-up, visible mold or dampness in the home, use of gas stove, birth season, parental socioeconomic status (low, medium, high), and intervention (GINIplus, MAAS, PIAMA). *p < 0.05.						

Effect estimates in two-pollutant models that included NO_2_ plus one of the PM exposures were closer to the null (vs. estimates from single-pollutant models), and the only statistically significant finding was for NO_2_ and otitis media (OR = 1.13; 95% CI: 1.01, 1.26 for a 10-μg/m^3^ increase in NO_2_) when adjusted for coarse PM (see Supplemental Material, Table S4). Confidence intervals increased substantially in two-pollutant models, reflecting the high correlation between pollutants (see Supplemental Material, Table S3).

All measures of air pollution were associated with pneumonia (*p* < 0.05) in analyses restricted to the first year of life (e.g., OR = 4.06; 95% CI: 1.93, 8.57 for a 5-μg/m^3^ increase in PM_2.5_) (Table 3). Further, the combined effect estimate for all associations (pneumonia, otitis media, and croup) increased when analyses were restricted to outcomes in the first year of life.

**Table 3:** Adjusted combined estimates for air pollution exposure at the birth address and respiratory infection by year of life [OR (95% CI)].

	Pneumonia^*a*^ (*n* = 12,891)	Otitis media^*b*^ (*n* = 8,722)	Croup^*c*^ (*n* = 9,101)
Respiratory infections during the first year^*d*^ of life
NO_2_	1.47* (1.15, 1.89)	1.19* (1.07, 1.33)	1.05 (0.83, 1.32)
NO_x_	1.45* (1.21, 1.75)	1.09 (0.98, 1.22)	1.10 (0.90, 1.36)
PM_2.5_	4.06* (1.93, 8.57)	1.21 (0.64, 2.28)	1.15 (0.67, 1.97)
PM_2.5_ absorbance	2.71* (1.68, 4.37)	1.32 (0.99, 1.75)	1.04 (0.59, 1.83)
PM_10_	1.77* (1.18, 2.67)	1.24 (0.76, 2.02)	1.07 (0.75, 1.53)
Coarse PM	1.46* (1.11, 1.92)	1.16 (0.80, 1.70)	1.02 (0.80, 1.30)
Traffic, nearest street	1.14* (1.07, 1.22)	0.99 (0.94, 1.04)	1.03 (0.94, 1.13)
Traffic, major streets	1.31* (1.15, 1.50)	1.03 (0.93, 1.14)	1.00 (0.81, 1.24)
Respiratory infections during the second year^*e*^ of life
NO_2_	1.40* (1.04, 1.88)	1.07 (0.96, 1.20)	0.92 (0.78, 1.09)
NO_x_	1.29* (1.07, 1.55)	1.02 (0.89, 1.17)	0.92 (0.78, 1.08)
PM_2.5_	2.65 (0.63, 11.2)	1.06 (0.64, 1.74)	0.76 (0.51, 1.15)
PM_2.5_ absorbance	1.90 (0.93, 3.87)	1.20 (0.80, 1.79)	0.89 (0.59, 1.35)
PM_10_	1.42 (0.99, 2.03)	1.00 (0.84, 1.19)	0.83 (0.63, 1.09)
Coarse PM	1.24 (0.98, 1.56)	1.00 (0.89, 1.13)	0.89 (0.73, 1.08)
Traffic, nearest street	1.05 (0.98, 1.13)	0.96 (0.90, 1.03)	0.93 (0.81, 1.07)
Traffic, major streets	1.10 (0.90, 1.34)	0.96 (0.83, 1.10)	1.00 (0.88, 1.14)
Associations are presented for the following increments in exposure: 10 μg/m^3^ for NO_2_, 20 μg/m^3^ for NO_x_, 5 μg/m^3^ for PM_2.5_, 1 unit for PM_2.5_ absorbance, 10 μg/m^3^ for PM_10_, 5 μg/m^3^ for coarse PM, 5,000 vehicles/day for traffic intensity on the nearest street; and 4,000 vehicle-km/day for traffic load on major roads within a 100-m buffer; associations with traffic intensity and traffic load were additionally adjusted for background NO_2_ concentrations.^***a***^Based on four studies: BAMSE, GINI/LISA North, GINI/LISA South, PIAMA. ^***b***^Based on 3 studies: BAMSE, LISAplus North, LISAplus South, PIAMA. ^***c***^Based on three studies: BAMSE, GINI/LISA North, GINI/LISA South. ^***d***^Defined as 0–12 months. ^***e***^Defined as 13–24 months. Models were adjusted for municipality (BAMSE), sex, older siblings, breastfeeding at 6 months, atopy of either parent, any child-care reported during follow-up, maternal smoking during pregnancy, any environmental tobacco smoke in the child’s home reported during follow-up, visible mold or dampness in the home, use of gas stove, birth season, parental socioeconomic status (low, medium, high), and intervention (GINIplus, PIAMA). *p < 0.05.			

Stratified meta-analyses suggested slightly stronger effects in females and in those from middle socioeconomic groups (see Supplemental Material, Tables S5 and S6). In analyses stratified by residential mobility during follow-up, the associations between air pollution and respiratory infection were not consistent by strata: Pneumonia effects were greater for movers (OR = 1.62; 95% CI: 1.20, 2.18 vs. 1.21; 95% CI: 0.88, 1.67 for NO_2_), whereas otitis media effects were greater for nonmovers (OR = 1.08; 95% CI: 1.01, 1.16 vs. 1.03; 95% CI: 0.71, 1.48 for NO_2_; see Supplemental Material, Table S7). Inclusion of additional covariates into the individual cohort models (birth weight, maternal age and area level socioeconomic indicators) did not change air pollution effect estimates or improve model fit (data not shown). There was no consistent evidence for spatial clustering when area-level variables were included as a random effect in models (data not shown). Finally, analyses using back-extrapolated monitoring data were generally consistent with the main findings (see Supplemental Material, Table S8).

## Discussion

As part of the ESCAPE project we had the unprecedented opportunity to examine outdoor air pollution as a risk factor for respiratory infection during early childhood in an analysis combining 10 European birth cohorts (*N*_Total_ = 16,059) with data on parent-reported physician-diagnosed pneumonia, otitis media, and croup; we also examined individual air pollution exposure estimates based on common ESCAPE protocols. We found consistent evidence for an association between air pollution and pneumonia, and some evidence for otitis media, during the first two years of life.

Urban air pollution has been associated with respiratory tract infections (Jedrychowski et al. 2013; Lin et al. 2005), pneumonia (Gouveia and Fletcher 2000), croup (Schwartz et al. 1991), persistent cough (Esplugues et al. 2011), and otitis media (MacIntyre et al. 2011) during childhood. Associations have also been reported for indoor air pollution and pneumonia in developing countries (da Costa et al. 2004; Mahalanabis et al. 2002) where concentrations are considerably higher than in our study areas. Our findings are consistent with previous studies that used similar methods to examine air pollution and otitis media in three of our cohorts—PIAMA (Brauer et al. 2006), LISAplus Munich (Brauer et al. 2006), and INMA (Aguilera et al. 2013)—and a recent meta-analysis on long-term PM_2.5_ and acute lower respiratory infection in children, which also included the PIAMA study (Mehta et al. 2013).

Similar to secondhand smoke (U.S. Department of Health and Human Services 2006), air pollution is thought to increase susceptibility to respiratory infections primarily via an inflammatory response (Li et al. 2008). Urban air pollution may impair defense mechanisms (Clarke et al. 2000; Leonardi et al. 2000), and oxidant pollutants, in particular, may exacerbate virus-induced inflammation of the respiratory system (Lambert et al. 2003; Spannhake et al. 2002).

Analyses were restricted to the first years of life to include the period of greatest age-specific incidence of respiratory infections (Schnabel et al. 2009; Walker et al. 2013). Our findings suggested that air pollution effects may be slightly stronger during the first year (Table 3). This finding could highlight a unique period of susceptibility when children are at increased risk of respiratory infections due to air pollution (Gehring et al. 2002; Gouveia and Fletcher 2000; Heinrich and Slama 2007). It is also possible that the null findings for infections during the second year of life are due to increased exposure misclassification as older children may spend less time at their home address due to increased child-care enrollment.

A unique strength of LUR models is their ability to capture small-scale spatial variability in exposure; however, the measurements used to create the ESCAPE exposure models were taken after the birth year (Eeftens et al. 2012a; Cyrys et al. 2012), and this may have introduced exposure misclassification. Although it is possible that overall levels of air pollution changed during this period, previous findings suggest that the spatial distribution of air pollutants within each area remained consistent (Cesaroni et al. 2012; Eeftens et al. 2011; Wang et al. 2013). Further, our sensitivity analyses using monitoring data to back-extrapolate exposure estimates to the actual first year of life were consistent with our main findings (see Supplemental Material, Table S8).

The wording of parental questionnaires was similar across each cohort, and previous research has shown good agreement between maternal recall and medical records during early childhood (D’Souza-Vazirani et al. 2005; Vernacchio et al. 2007). Geographic differences in the prevalence of outcomes across the cohorts were most pronounced for otitis media and may point to potential diagnostic biases or disease misclassification between countries. It was not possible to adjust for epidemics, the impact of vaccinations, or the frequency of infections because data were not available across all cohorts. Furthermore, defining upper respiratory tract infections (otitis media, croup) by physician diagnosis is complicated by the fact that not all infections present with acute symptoms severe enough to warrant a physician visit, in contrast with pneumonia, which routinely presents with a high fever and/or difficulty breathing (Edmond et al. 2012).

## Conclusion

Our meta-analysis of 10 European birth cohorts found consistent evidence for an association between traffic-related air pollution and pneumonia, and some evidence to suggest an association with otitis media. Policies aimed at reducing air pollution may be successful in reducing the overall burden of pneumonia in early childhood.

## Correction

The value for “Traffic load on major streets within 100-m buffer” for GINI/LISA South in Table 1 was incorrect in the manuscript originally published online. It has been corrected here.

## Supplemental Material

(1.2 MB) PDF
