# Correction: Air Pollution and Respiratory Infections during Early Childhood: An Analysis of 10 European Birth Cohorts within the ESCAPE Project

**DOI:** 10.1289/ehp.1306755-erratum

**Published:** 2013-10-22

The value for “Traffic load on major streets within 100‑m buffer” for GINI/LISA South in Table 1 was incorrect in the manuscript originally published online. It has been corrected here.

