# Supplementary material for: Air Pollution and Respiratory Infections during Early Childhood: An Analysis of 10 European Birth Cohorts within the ESCAPE Project
Source: Environ Health Perspect. 2013 Oct 22;122(1):107–13. doi: 10.1289/ehp.1306755 (PMC3888562; doi:10.1289/ehp.1306755)
Supplement: (1.2 MB) PDF [file ehp.1306755.s001.508.pdf]

Supplemental Material

Air Pollution and Respiratory Infections during Early Childhood: An Analysis of 10 European Birth Cohorts within the ESCAPE Project

Elaina A. MacIntyre, Ulrike Gehring, Anna Mölter, Elaine Fuertes, Claudia Klümper, Ursula Krämer, Ulrich Quass, Barbara Hoffmann, Mireia Gascon, Bert Brunekreef, Gerard H. Koppelman, Rob Beelen, Gerard Hoek, Matthias Birk, Johan C. de Jongste, H.A. Smit, Josef Cyrys, Olena Gruzieva, Michal Korek, Anna Bergström, Raymond M. Agius, Frank de Vocht, Angela Simpson, Daniela Porta, Francesco Forastiere, Chiara Badaloni, Giulia Cesaroni, Ana Esplugues, Ana Fernández-Somoano, Aitana Lerxundi, Jordi Sunyer, Marta Cirach, Mark J. Nieuwenhuijsen, Göran Pershagen, and Joachim Heinrich

Table of Contents

Specific wording of parental administered questionnaires - 2 -

Table S1. Distribution of adjustment covariates, by cohort - 3 -

Table S2. Air pollution distributions, by cohort - 5 -

Table S3. Correlations between pollutants, by cohort - 7 -

Table S4. Combined estimates using two-pollutant models -9 -

Table S5. Combined estimates stratified by gender -10 -

Table S6. Combined estimates stratified by parental socio-economic status -11 -

Table S7. Combined estimates stratified by residential mobility -12 -

Table S8. Combined estimates using back-extrapolated air pollution data -13-

Specific wording of parental administered questionnaires

BAMSE

Asked at 12 months
Has a doctor ever diagnosed your child as having [pneumonia/croup or pseudocroup]?
Has your child had earaches that were treated with antibiotics (e.g., penicillin)?

Asked at 24 months
Has a doctor diagnosed your child as having [pneumonia/croup or pseudocroup] after the age of one year?
Has your child had earaches, ear infections with fluid behind the eardrum after the age of one year? (with antibiotic treatment)

GASPII

Asked at 6 and 15 months
Has your child had any of the following illnesses? (oneumonia, otitis media)

GINIplus

Asked at 12 and 24 months
Has your child received a physician diagnosis of [pneumonia/croup] during the past 12 months?

INMA

Asked at 12 (Valencia), 14 (Gipuzkoa and Sabadell) and 18 (Asturias) months
Has a doctor told you that your son/daughter has had [otitis/pneumonia]?

LISAplus

Asked at 6, 12, 18 and 24 months
Has your child received a physician diagnosis of [pneumonia/otitis media/croup] during the past 6 months?

MAAS

Asked at 3 years
Has your child had any of the following illnesses? (Pneumonia, Croup)

PIAMA

Asked at 12 and 24 months
Did a doctor diagnose [infection of the middle ear/pneumonia] in your child in the last 12 months?

Supplemental Material, Table S1. Distribution of adjustment covariates, by cohort.

	BAMSE N (%)	GASPII N (%)	GINI/LISA South N (%)	GINI/LISA North N (%)	INMA Asturias N (%)	INMA Gipuzkoa N (%)	INMA Sabadell N (%)	INMA Valencia N (%)	MAAS N (%)	PIAMA N (%)
Total number of children	3821 (100)	678 (100)	3321 (100)	2460 (100)	360 (100)	437 (100)	402 (100)	559 (100)	695 (100)	3475 (100)
Male gender	1943 (50.8)	342 (50.4)	1739 (52.4)	1266 (51.5)	186 (51.7)	217 (49.7)	214 (53.2)	293 (52.4)	376 (55.1)	1788 (51.5)
Older siblings	1835 (48.0)	270 (39.8)	1368 (41.2)	1319 (53.6)	140 (38.9)	196 (44.9)	166 (41.3)	244 (43.7)	340 (48.9)	1753 (50.5)
Breastfeeding at 6 months	1833 (48.0)	361 (53.2)	1773 (53.4)	972 (39.5)	217 (60.3)	352 (80.6)	323 (80.4)	368 (65.8)	212 (30.5)	891 (25.6)
Parental atopy	1131 (29.6)	186 (27.4)	2447 (74.6)	1475 (60.0)	140 (38.9)	176 (40.3)	206 (51.2)	225 (40.3)	576 (82.9)	1747 (50.3)
Maternal smoking during pregnancy	482 (12.6)	82 (12.1)	430 (13.0)	408 (16.6)	106 (29.4)	109 (24.9)	122 (30.4)	239 (42.8)	74 (10.6)	577 (16.6)
Tobacco smoke in the home at 1yr	436 (11.4)	208 (30.7)[b]	569 (17.1)	573 (23.3)	-	-	-	-	295 (42.4)	1079 (31.1)
Tobacco smoke in the home at 2yrs	511 (13.4)	-	835 (25.1)	996 (40.5)	-	-	-	-	-	1158 (33.3)
Daycare at 1yr	-	178 (26.3)[b]	205 (6.3)	41 (1.7)	177 (49.2)	218 (49.9)	122 (30.3)	114 (20.4)	-	834 (24.1)
Daycare at 2yrs	2664 (72.1)	-	474 (14.3)	61 (2.5)	-	-	-	-	493 (70.9)	1032 (29.7)
Socio-economic status[a]										
Low	87 (2.3)	61 (9.0)	178 (5.4)	334 (13.6)	33 (9.2)	44 (10.1)	40 (10.0)	32 (5.7)	105 (15.1)	438 (12.6)
Middle	1688 (44.2)	297 (43.8)	589 (17.7)	980 (39.8)	57 (15.8)	85 (19.5)	74 (18.4)	94 (16.8)	273 (39.3)	1273 (36.6)
High	2044 (53.5)	320 (47.2)	2554 (76.9)	1146 (46.6)	270 (75.0)	308 (70.5)	288 (71.6)	433 (77.5)	201 (28.9)	1764 (50.8)
Very high[b]									116 (16.7)	
Municipality/region[c]										
1	1143 (29.9)									1104 (31.8)
2	1102 (28.8)									1422 (40.9)

	BAMSE N (%)	GASPII N (%)	GINI/LISA South N (%)	GINI/LISA North N (%)	INMA Asturias N (%)	INMA Gipuzkoa N (%)	INMA Sabadell N (%)	INMA Valencia N (%)	MAAS N (%)	PIAMA N (%)
3	1003 (26.3)									949 (27.3)
4	573 (15.0)									
Intervention arm	-	-	902 (43.4)	782 (35.7)	-	-	-	-	-	319 (9.2)
Gas stove in the home	438 (11.5)	672 (99.1)	289 (8.7)	116 (4.7)	69 (19.2)	66 (15.1)	251 (62.4)	352 (63.0)	538 (77.5)	2909 (83.7)
Visible mold or dampness in the home	967 (25.3)	110 (16.2)	1174 (35.4)	536 (21.8)	74 (20.6)	55 (12.6)	67 (16.7)	46 (8.2)	123 (17.7)	1744 (50.2)
Maternal age under 30 years at time of birth	1953 (51.1)	188 (27.7)	1113 (33.5)	1259 (51.2)	151 (41.9)	200 (45.8)	212 (52.7)	311 (55.6)	223 (36.1)	1788 (51.5)
Birth season										
Winter (Jan-Mar)	677 (17.7)	126 (18.6)	996 (30.1)	741 (30.1)	76 (21.2)	124 (28.4)	92 (22.9)	145 (25.9)	207 (29.8)	642 (18.5)
Spring (Apr-Jun)	1124 (29.4)	233 (34.4)	886 (26.7)	689 (28.0)	100 (27.8)	84 (19.2)	80 (19.9)	186 (33.3)	193 (27.8)	906 (26.1)
Summer (Jul-Sep)	1109 (29.0)	189 (27.9)	764 (23.0)	555 (22.6)	120 (33.3)	110 (25.2)	92 (22.9)	124 (22.2)	159 (22.9)	1080 (31.1)
Fall (Oct-Dec)	911 (23.9)	130 (19.2)	675 (20.3)	475 (19.3)	64 (17.8)	119 (27.2)	138 (34.3)	104 (18.6)	136 (19.6)	847 (24.4)
Residential mobility at anytime during follow-up	1411 (36.9)	69 (10.2)	959 (30.6)	511 (20.8)	52 (15.0)	21 (4.9)	39 (10.0)	51 (9.2)	138 (21.2)	588 (17.0)

[a] Highest education attained by either parent (BAMSE, GINI/LISA North, GINI/LISA South, PIAMA, INMA); highest occupational level by either parent (GASPII); or household income (MAAS: less than £10,000; £10,000-20,000; £20,000-30,000; more than £30,000); [b] MAAS only; [c] BAMSE municipality: 1- Stockholm, 2- Jarfalla, 3- Solna, 4- Sundbyberg; PIAMA region: 1-North, 2-Middle, 3-West.

Supplemental Material, Table S2. Air pollution distributions, by cohort.

	**BAMSE**	**GASPII**	**GINI/LISA South**	**GINI/LISA North**	**INMA Asturias**	**INMA Gipuzkoa**	**INMA Sabadell**	**INMA Valencia**	**MAAS**	**PIAMA**
NO_2 (µg/m^3)										
Mean ± SD	14.1±5.4	44.6±9.9	21.8±6.1	23.7±3.3	23.4±10.6	18.7±4.3	41.7±10.6	27.4±11.8	23.0±2.0	23.0±6.6
Median	12.4	43.2	20.8	23.2	22.2	18.4	41.7	27.9	23.0	23.1
IQR	9.3	10.4	8.3	3.2	14.4	5.7	12.4	18.1	2.4	8.4
Range (Min-Max)	6.0-33.0	22.5-85.1	11.5-61.1	19.7-62.8	0.0-52.6	9.8-32.3	17.8-87.5	2.6-72.9	16.0-30.4	8.7-59.6
NO_x (µg/m^3)										
Mean ± SD	25.6±12.0	70.4±20.6	36.3±10.4	34.6±9.0	47.4±28.0	37.9±8.4	71.9±23.9	44.2±21.9	38.9±4.9	34.0±11.5
Median	20.9	65.8	34.6	33.3	44.0	37.2	69.7	44.7	38.7	32.8
IQR	18.2	23.9	12.2	8.4	39.0	11.1	21.2	31.1	5.2	11.0
Range (Min-Max)	11.5-86.3	25.7-154.7	19.7-121.4	23.9-147.7	0.0-128.9	20.6-64.9	28.9-208.0	0.7-140.8	26.1-77.8	16.5-98.9
$PM_{2.5}$ (µg/m^3)										
Mean ± SD	7.8±8.1	19.5±2.1	13.4±1.0	17.4±0.7	-	-	14.6±1.3	-	9.5±0.2	16.4±0.7
Median	8.1	18.8	13.3	17.2	-	-	14.6	-	9.4	16.5
IQR	1.9	2.0	1.2	0.9	-	-	1.1	-	0.0	1.2
Range (Min-Max)	4.2-11.4	17.0-27.4	10.6-18.3	15.8-21.5	-	-	11.0-20.5	-	9.4-11.0	15.3-21.1
$PM_{2.5}$ absorbance (10^{-5}/m)										
Mean ± SD	0.7±0.2	2.7±0.6	1.7±0.2	1.2±0.2	-	-	2.2±0.4	-	1.1±0.2	1.2±0.2
Median	0.6	2.5	1.7	1.2	-	-	2.2	-	1.1	1.2
IQR	0.3	0.4	0.2	0.2	-	-	0.5	-	0.2	0.3
Range (Min-Max)	0.4-1.3	2.2-4.8	1.3-3.6	1.0-3.1	-	-	1.4-4.3	-	0.7-2.0	0.8-3.0
PM_{10} (µg/m^3)										
Mean ± SD	15.7±3.9	36.9±5.7	20.4±2.4	25.5±1.2	-	-	27.0±3.4	-	17.1±0.9	24.9±1.2
Median	15.6	34.9	20.4	25.2	-	-	26.4	-	17.0	24.6
IQR	3.9	6.1	2.9	1.6	-	-	3.4	-	0.2	1.2
Range (Min-Max)	6.0-30.9	29.7-58.2	14.8-34.4	23.9-33.9	-	-	21.8-39.0	-	12.7-22.7	23.7-33.2
Coarse PM (µg/m^3)										
Mean ± SD	7.9±3.0	16.8±3.9	6.8±1.5	8.5±0.7	-	-	11.5±2.3	-	7.0±0.9	8.4±0.8
Median	7.7	15.7	6.5	8.4	-	-	11.3	-	6.9	8.1
IQR	3.0	4.3	2.0	0.7	-	-	2.5	-	0.8	0.8
Range (Min-Max)	0.7-20.2	10.8-31.5	4.1-16.0	1.9-13.8	-	-	8.6-20.6	-	5.0-11.5	7.6-14.0

	BAMSE	GASPII	GINI/LISA South	GINI/LISA North	INMA Asturias	INMA Gipuzkoa	INMA Sabadell	INMA Valencia	MAAS	PIAMA
Traffic intensity on nearest street (vehicles/day)										
Mean ± SD	2418±4593	-	2711±7989	1188±2600	-	-	-	-	814±2257	1053±3301
Median	500	-	500	500	-	-	-	-	500	215
IQR	1450	-	0	0	-	-	-	-	0	436
Range (Min-Max)	122-52020	-	500-134000	500-27522	-	-	-	-	500-29590	0-54610
Traffic load on all major roads (within 100m) (vehicle*m/day)										
Mean ± SD	1024574±1783254	-	1087791±3116387	284605±798545	-	-	-	-	710648±3433417	542073±1488584
Median	0	-	0	0	-	-	-	-	0	0
IQR	1621333	-	1318179	0	-	-	-	-	0	0
Range (Min-Max)	0-26100000	-	0-52741070	0-11178024	-	-	-	-	0-63463780	0-23498579

Supplemental Material, Table S3. Correlations between pollutants, by cohort.

	NO_x	$PM_{2.5}$	$PM_{2.5}$ absorbance	PM_{10}	Coarse PM	Traffic, nearest	Traffic, major
**BAMSE**							
NO_2	.96	.74	.92	.63	.64	.56	.45
NO_x		.67	.82	.62	.63	.68	.45
$PM_{2.5}$			.79	.77	.73	.38	.42
$PM_{2.5}$ absorbance				.96	.91	.44	.51
PM_{10}					.95	.54	.52
Coarse PM						.56	.54
Traffic, nearest							.51
**GASPII**							
NO_2	.76	.67	.52	.62	.76		
NO_x		.72	.61	.69	.76		
$PM_{2.5}$			.78	.95	.92		
$PM_{2.5}$ absorbance				.74	.72		
PM_{10}					.94		
**GINI/LISA South**							
NO_2	.95	.43	.75	.68	.93	.45	.53
NO_x		.58	.84	.72	.91	.52	.62
$PM_{2.5}$			.59	.47	.43	.38	.43
$PM_{2.5}$ absorbance				.72	.82	.51	.63
PM_{10}					.76	.28	.34
Coarse PM						.38	.47
Traffic, nearest							.68
**GINI/LISA North**							
NO_2	.98	.72	.75	.72	.58	.26	.46
NO_x		.72	.68	.71	.56	.25	.33
$PM_{2.5}$			.72	.83	.63	.14	.24
$PM_{2.5}$ absorbance				.83	.65	.36	.65
PM_{10}					.68	.13	.26
Coarse PM						.15	.28

	NO_x	$PM_{2.5}$	$PM_{2.5}$ absorbance	PM_{10}	Coarse PM	Traffic, nearest	Traffic, major
Traffic, nearest							.58
**INMA Sabadell**							
NO_2	.96	.80	.93	.81	.52		
NO_x		.83	.93	.84	.61		
$PM_{2.5}$			.85	.78	.63		
$PM_{2.5}$ absorbance				.80	.55		
PM_{10}					.87		
**MAAS**							
NO_2	.66	.42	.40	.36	.34	.19	.12
NO_x		.26	.50	.31	.29	.29	.54
$PM_{2.5}$			.19	.27	.26	.00	.00
$PM_{2.5}$ absorbance				.44	.53	.04	.26
PM_{10}					.73	.05	.11
Coarse PM						.04	.02
Traffic, nearest							.11
**PIAMA**							
NO_2	.88	.72	.90	.80	.76	.25	.37
NO_x		.73	.89	.87	.76	.30	.43
$PM_{2.5}$			.88	.87	.76	.28	.37
$PM_{2.5}$ absorbance				.91	.77	.33	.46
PM_{10}					.80	.29	.48
Coarse PM						.38	.40
Traffic, nearest							.48

Supplemental Material, Table S4. Combined estimates using two-pollutant models.

	Pneumonia		Otitis Media		Croup	
	N	OR (95% CI)[a]	N	OR (95% CI)	N	OR (95% CI)
**Single pollutant models**						
NO_2	16059	1.30* (1.02, 1.65)	11094	1.09* (1.02, 1.16)	10170	0.96 (0.83, 1.12)
$PM_{2.5}$	14009	2.58 (0.91, 7.27)	9738	1.06 (0.75, 1.49)	10170	0.90 (0.63, 1.30)
PM_{10}	14703	1.76* (1.00, 3.09)	9738	0.98 (0.84, 1.14)	10170	0.92 (0.72, 1.18)
Coarse PM	14703	1.24* (1.03, 1.50)	9738	0.97 (0.88, 1.08)	10170	0.97 (0.82, 1.15)
$PM_{2.5}$ absorbance	14703	1.99* (1.44, 2.75)	9738	1.08 (0.83, 1.39)	10170	1.03 (0.72, 1.47)
**Two pollutant models**						
Model 1						
NO_2	14703	1.16 (0.84, 1.59)	9738	1.06 (0.96, 1.18)	10170	0.98 (0.82, 1.17)
$PM_{2.5}$	14703	1.91 (0.56, 6.57)	9738	0.94 (0.70, 1.28)	10170	0.89 (0.57, 1.39)
Model 2						
NO_2	14703	1.28 (0.99, 1.66)	9738	1.08 (0.96, 1.22)	10170	1.01 (0.82, 1.23)
PM_{10}	14703	1.10 (0.78, 1.56)	9738	0.91 (0.76, 1.10)	10170	0.91 (0.64, 1.31)
Model 3						
NO_2	14703	1.38 (0.93, 2.05)	9738	1.13* (1.01, 1.26)	10170	1.01 (0.78, 1.31)
Coarse PM	14703	1.13 (0.72, 1.76)	9738	0.91 (0.80, 1.04)	10170	0.97 (0.78, 1.21)
Model 4						
NO_2	14703	1.18 (0.74, 1.91)	9738	1.06 (0.93, 1.21)	10170	0.94 (0.75, 1.17)
$PM_{2.5}$ absorbance	14703	1.57 (0.81, 3.04)	9738	0.97 (0.67, 1.41)	10170	1.23 (0.73, 2.07)

[a] Individual cohort models were adjusted for municipality (BAMSE), gender, older siblings, breastfeeding at 6 months, atopy of either parent, any daycare reported during follow-up, maternal smoking during pregnancy, any environmental tobacco smoke in the child's home reported during follow-up, visible mold or dampness in the home, gas stove, birth season, parental socio-economic status (low, medium, high), and intervention (GINIplus, MAAS, PIAMA).

*Statistically significant elevated odds ratios ($p < 0.05$). Associations are presented for the following increments in exposure: 10 µg/m³ for NO_2, 5 µg/m³ for $PM_{2.5}$, 10 µg/m³ for PM_{10}, 1 unit for $PM_{2.5}$ absorbance, 5 µg/m³ for coarse PM.

Supplemental Material, Table S5. Combined estimates stratified by gender.

	Pneumonia				Otitis Media				Croup			
	Female		Male		Female		Male		Female		Male	
	N	OR (95% CI)	N	OR (95% CI)	N	OR (95% CI)	N	OR (95% CI)	N	OR (95% CI)	N	OR (95% CI)
NO_2	7771	1.40* (1.13, 1.74)	8288	1.37 (0.97, 1.93)	5385	1.11 (0.96, 1.29)	5709	1.07 (0.98, 1.17)	4911	0.97 (0.76, 1.24)	5259	0.96 (0.79, 1.17)
NO_x	7771	1.30* (1.07, 1.58)	8288	1.38* (1.00, 1.91)	5385	1.07 (0.94, 1.22)	5709	1.03 (0.95, 1.13)	4911	1.01 (0.81, 1.27)	5259	0.98 (0.81, 1.17)
$PM_{2.5}$	6605	2.52 (0.82, 7.75)	7216	2.82 (0.89, 8.87)	4725	1.03 (0.62, 1.71)	5013	1.1 (0.73, 1.66)	4592	0.93 (0.52, 1.66)	4883	0.89 (0.52, 1.53)
$PM_{2.5}$ absorbance	6605	2.26* (1.38, 3.71)	7216	1.86* (1.20, 2.90)	4725	1.12 (0.84, 1.49)	5013	1.08 (0.77, 1.52)	4592	1.04 (0.58, 1.86)	4883	0.92 (0.57, 1.48)
PM_{10}	6605	1.93* (1.14, 3.26)	7216	1.50 (0.69, 3.26)	4725	0.99 (0.80, 1.23)	5013	0.98 (0.79, 1.21)	4592	0.94 (0.63, 1.4)	4883	0.91 (0.66, 1.25)
Coarse PM	6923	1.47* (1.12, 1.94)	7592	1.10 (0.84, 1.43)	4725	0.96 (0.83, 1.12)	5013	0.98 (0.85, 1.13)	4911	0.93 (0.71, 1.22)	5259	0.98 (0.79, 1.22)
Traffic, nearest	6587	1.03 (0.90, 1.17)	7036	1.11* (1.04, 1.17)	4201	0.99 (0.83, 1.17)	4457	0.98 (0.93, 1.03)	4911	0.97 (0.86, 1.09)	5259	1.00 (0.94, 1.07)
Traffic, major	6587	1.19 (0.95, 1.50)	7036	1.21* (1.07, 1.37)	4201	0.93 (0.78, 1.1)	4457	1.01 (0.9, 1.12)	4911	0.99 (0.79, 1.25)	5259	0.99 (0.86, 1.15)

Individual cohort models were adjusted for municipality (BAMSE), gender, older siblings, breastfeeding at 6 months, atopy of either parent, any daycare reported during follow-up, maternal smoking during pregnancy, any environmental tobacco smoke in the child's home reported during follow-up, visible mold or dampness in the home, gas stove, birth season, parental socio-economic status (low, medium, high), and intervention (GINIplus, MAAS, PIAMA).

*Statistically significant elevated odds ratios ($p < 0.05$). Associations are presented for the following increments in exposure: 10 µg/m³ for NO_2, 20 µg/m³ for NO_x, 5 µg/m³ for $PM_{2.5}$, 1 unit for $PM_{2.5}$ absorbance, 10 µg/m³ for PM_{10}, 5 µg/m³ for coarse PM, 5,000 vehicles/day for traffic intensity on the nearest street; and 4,000,000 vehicle·m/day for traffic load on major roads within a 100 m buffer; associations with traffic intensity and traffic load were additionally adjusted for background NO_2 concentrations.

Supplemental Material, Table S6. Combined estimates stratified by parental socio-economic status.

		Low		Middle		High
	N	OR (95% CI)	N	OR (95% CI)	N	OR (95% CI)
**Pneumonia**						
NO_2	1212	1.03 (0.55, 1.93)	5021	1.54 (1.18, 2.01)	9174	1.32 (1.00, 1.70)
NO_x	1212	0.82 (0.41, 1.64)	5021	1.57 (1.23, 1.99)	9174	1.27 (0.99, 1.62)
$PM_{2.5}$	1136	0.3 (0.03, 2.84)	4818	3.22 (1.19, 8.71)	9174	2.63 (0.81, 8.47)
$PM_{2.5}$ absorbance	1136	0.77 (0.15, 3.91)	4818	2.33 (1.28, 4.23)	9174	2.08 (1.37, 3.15)
PM_{10}	1136	0.32 (0.04, 2.57)	4818	1.65 (1.01, 2.71)	9174	1.68 (0.82, 3.43)
Coarse PM	1136	0.51 (0.12, 2.15)	4818	1.36 (0.97, 1.9)	9174	1.22 (0.90, 1.65)
Traffic, nearest	1136	1.41 (0.95, 2.08)	4447	1.18 (1.05, 1.32)	9174	1.05 (0.98, 1.14)
Traffic, major	1136	1.58 (0.63, 3.97)	4447	1.43 (1.14, 1.81)	9174	1.09 (0.86, 1.38)
**Otitis Media**						
NO_2	813	0.92 (0.64, 1.32)	3767	1.21 (0.99, 1.47)	6284	1.07 (0.95, 1.19)
NO_x	813	0.84 (0.63, 1.12)	3767	1.11 (0.9, 1.37)	6284	1.05 (0.96, 1.16)
$PM_{2.5}$	697	0.35 (0.06, 1.96)	3555	1.06 (0.62, 1.83)	5486	1.16 (0.78, 1.74)
$PM_{2.5}$ absorbance	697	0.3 (0.05, 1.77)	3555	1.18 (0.85, 1.63)	5486	1.09 (0.81, 1.48)
PM_{10}	697	0.69 (0.24, 1.97)	3555	0.94 (0.66, 1.34)	5486	1.06 (0.88, 1.29)
Coarse PM	697	0.85 (0.42, 1.73)	3555	0.9 (0.76, 1.07)	5486	1.02 (0.9, 1.17)
Traffic, nearest	596	0.76 (0.52, 1.12)	3184	1.05 (0.94, 1.16)	4878	0.95 (0.9, 1.01)
Traffic, major	596	1.19 (0.62, 2.28)	3184	1.06 (0.88, 1.27)	4878	0.95 (0.81, 1.12)
**Croup**						
NO_2	703	0.37 (0.16, 0.87)	3454	1.03 (0.74, 1.43)	6013	1.00 (0.84, 1.20)
NO_x	703	0.42 (0.19, 0.93)	3454	1.09 (0.83, 1.45)	6013	1.02 (0.86, 1.21)
$PM_{2.5}$	703	0.64 (0.11, 3.91)	3454	1.36 (0.69, 2.66)	6013	0.82 (0.44, 1.54)
$PM_{2.5}$ absorbance	703	0.72 (0.11, 4.59)	3454	1.21 (0.61, 2.37)	6013	1.05 (0.64, 1.74)
PM_{10}	703	0.29 (0.06, 1.47)	3454	1.22 (0.78, 1.91)	6013	0.87 (0.58, 1.32)
Coarse PM	703	0.82 (0.13, 5.24)	3454	1.03 (0.76, 1.40)	6013	0.98 (0.74, 1.28)
Traffic, nearest	703	1.04 (0.72, 1.50)	3454	0.97 (0.84, 1.11)	6013	1.00 (0.94, 1.07)
Traffic, major	703	0.80 (0.12, 5.15)	3454	1.20 (0.90, 1.60)	6013	0.94 (0.75, 1.17)

Individual cohort models were adjusted for municipality (BAMSE), gender, older siblings, breastfeeding at 6 months, atopy of either parent, any daycare reported during follow-up, maternal smoking during pregnancy, any environmental tobacco smoke in the child's home reported during follow-up, visible mold or dampness in the home, gas stove, birth season, and intervention (GINIplus, MAAS, PIAMA). Associations are presented for the following increments in exposure: 10 $\mu g/m^3$ for NO_2, 20 $\mu g/m^3$ for NO_x, 5 $\mu g/m^3$ for $PM_{2.5}$, 1 unit for $PM_{2.5}$ absorbance, 10 $\mu g/m^3$ for PM_{10}, 5 $\mu g/m^3$ for coarse PM, 5,000 vehicles/day for traffic intensity on the nearest street; and 4,000,000 vehicle·m/day for traffic load on major roads within a 100 m buffer; associations with traffic intensity and traffic load were additionally adjusted for background NO_2 concentrations.

Supplemental Material, Table S7. Combined estimates stratified by residential mobility.

	**Pneumonia**				**Otitis Media**				**Croup**			
		**Movers**		**Non-movers**		**Movers**		**Non-movers**		**Movers**		**Non-movers**
	**N**	**OR (95% CI)**	**N**	**OR (95% CI)**	**N**	**OR (95% CI)**	**N**	**OR (95% CI)**	**N**	**OR (95% CI)**	**N**	**OR (95% CI)**
NO_2	3796	1.62* (1.20, 2.18)	12098	1.21 (0.88, 1.67)	2545	1.03 (0.71, 1.48)	8427	1.08* (1.01, 1.16)	2985	0.82 (0.62, 1.1)	7139	1.03 (0.86, 1.24)
NO_x	3796	1.51* (1.16, 1.97)	12098	1.17 (0.91, 1.50)	2545	1.02 (0.76, 1.36)	8427	1.04 (0.97, 1.12)	2985	0.89 (0.69, 1.16)	7139	1.05 (0.88, 1.24)
$PM_{2.5}$	3495	4.11* (1.42, 11.9)	10443	2.10 (0.70, 6.36)	2421	0.94 (0.35, 2.49)	7286	1.07 (0.83, 1.38)	2847	0.92 (0.32, 2.61)	6624	0.92 (0.59, 1.44)
$PM_{2.5}$ absorbance	3495	3.6* (1.83, 7.1)	10443	1.66* (1.10, 2.50)	2421	1.08 (0.65, 1.8)	7286	1.08 (0.82, 1.42)	2847	0.64 (0.31, 1.32)	6624	1.15 (0.75, 1.77)
PM_{10}	3495	2.41 (0.81, 7.16)	10443	1.53 (0.82, 2.87)	2421	0.99 (0.52, 1.85)	7286	1.00 (0.84, 1.2)	2847	0.75 (0.49, 1.14)	6624	1.02 (0.75, 1.39)
Coarse PM	3633	2.12 (0.89, 5.03)	10957	1.24 (0.98, 1.57)	2421	0.88 (0.51, 1.52)	7286	1.01 (0.89, 1.14)	2985	0.76 (0.58, 1.01)	7139	1.11 (0.90, 1.38)
Traffic, nearest	3564	1.17* (1.07, 1.28)	9988	1.06 (0.98, 1.14)	2313	1.00 (0.93, 1.08)	6317	0.95 (0.90, 1.01)	2985	0.97 (0.85, 1.1)	7139	1.00 (0.91, 1.09)
Traffic, major	3564	1.30* (1.10, 1.54)	9988	1.12 (0.95, 1.32)	2313	1.02 (0.88, 1.19)	6317	0.96 (0.8, 1.14)	2985	0.85 (0.64, 1.11)	7139	1.07 (0.94, 1.23)

Individual cohort models were adjusted for municipality (BAMSE), gender, older siblings, breastfeeding at 6 months, atopy of either parent, any daycare reported during follow-up, maternal smoking during pregnancy, any environmental tobacco smoke in the child's home reported during follow-up, visible mold or dampness in the home, gas stove, birth season, parental socio-economic status (low, medium, high), and intervention (GINIplus, MAAS, PIAMA).

*Statistically significant elevated odds ratios ($p < 0.05$). Associations are presented for the following increments in exposure: 10 µg/m³ for NO_2, 20 µg/m³ for NO_x, 5 µg/m³ for $PM_{2.5}$, 1 unit for $PM_{2.5}$ absorbance, 10 µg/m³ for PM_{10}, 5 µg/m³ for coarse PM, 5,000 vehicles/day for traffic intensity on the nearest street; and 4,000,000 vehicle·m/day for traffic load on major roads within a 100 m buffer; associations with traffic intensity and traffic load were additionally adjusted for background NO_2 concentrations.

Table S8. Combined estimates using back-extrapolated air pollution data.

	Pneumonia		Otitis Media		Croup	
	N	OR (95% CI)	N	OR (95% CI)	N	OR (95% CI)
Difference method						
NO_2	16059	1.33 (0.99, 1.78)	11115	1.05 (0.97, 1.14)	10170	0.98 (0.85, 1.14)
NO_x	16059	1.31* (1.05, 1.63)	11115	1.02 (0.95, 1.10)	10170	1.01 (0.88, 1.16)
PM_{10}	14669	1.63 (0.99, 2.69)	9704	1.00 (0.86, 1.15)	10115	0.91 (0.72, 1.16)
Ratio method						
NO_2	16059	1.25 (0.99,1.58)	11115	1.05 (0.99, 1.12)	10170	1.00 (0.89, 1.14)
NO_x	16059	1.23* (1.02, 1.49)	11115	1.01 (0.96, 1.06)	10170	1.02 (0.93, 1.11)
PM_{10}	14669	1.57* (1.00, 2.47)	9704	0.99 (0.87, 1.12)	10115	0.92 (0.76, 1.12)

Individual cohort models were adjusted for municipality (BAMSE), gender, older siblings, breastfeeding at 6 months, atopy of either parent, any daycare reported during follow-up, maternal smoking during pregnancy, any environmental tobacco smoke in the child's home reported during follow-up, visible mold or dampness in the home, gas stove, birth season, parental socio-economic status (low, medium, high), and intervention (GINIplus, MAAS, PIAMA). *Statistically significant elevated odds ratios ($p < 0.05$). Associations are presented for the following increments in exposure: 10 µg/m³ for NO_2, 20 µg/m³ for NO_x, 10 µg/m³ for PM_{10}.
